# Long‐term outcome of definitive radiotherapy for locally advanced non‐small cell lung cancer: A real‐world single‐center study in the pre‐durvalumab era

**DOI:** 10.1002/cam4.70051

**Published:** 2024-07-31

**Authors:** Hong Zhu, Yi Xu, Huiquan Gao, Xingwen Fan, Ming Fan, Kuaile Zhao, Huanjun Yang, Zhengfei Zhu, Kailiang Wu

**Affiliations:** ^1^ Department of Radiation Oncology Fudan University Shanghai Cancer Center Shanghai China; ^2^ Department of Radiation Oncology, Tongji Hospital Tongji University School of Medicine Shanghai China; ^3^ Department of Oncology Shanghai Medical College, Fudan University Shanghai China; ^4^ Shanghai Clinical Research Center for Radiation Oncology Shanghai China; ^5^ Shanghai Key Laboratory of Radiation Oncology Shanghai China

**Keywords:** chemoradiotherapy, definitive radiotherapy, locally advanced non‐small cell lung cancer, prognostic factors, treatment outcome

## Abstract

**Background:**

There was limited research data on large‐scale locally advanced non‐small cell lung cancer (LA‐NSCLC) radical radiotherapy (RT) reported in China. This study examined overall survival (OS), progression‐free survival (PFS), treatment effectiveness, and toxicity in patients with LA‐NSCLC treated with definitive RT in the pre‐durvalumab era.

**Methods:**

A retrospective analysis of demographic information, clinical characteristics, treatment patterns, and clinical outcomes of 789 patients with LA‐NSCLC who underwent radical RT at our center between January 2005 and December 2015 was performed. The Kaplan–Meier method and log‐rank test were used for survival comparisons, and Cox regression was used for multivariate analysis.

**Results:**

There were 328 patients with stage IIIA disease and 461 with stage IIIB disease. By the last follow‐up, there were 365 overall deaths and 576 cases of recurrence, metastasis, or death. The median survival time was 31 months. The OS rates at 1, 2, 5, and 10 years were 83.7%, 59.5%, 28.8%, and 18.9%, respectively. PFS rates at 1, 2, 5, and 10 years were 48%, 24.5%, 11.9%, and 5.5%, respectively. Rates of ≥grade 3 acute radiation pneumonitis or esophagitis were 7.6% and 1.9%, respectively. Rates of ≥grade 3 chronic radiation pneumonitis and esophagitis were 11% and 0.4%, respectively. Multivariate analysis showed that the Karnofsky Performance Status (KPS) score, smoking status, and combined chemotherapy were prognostic factors for OS (*p <* 0.05). Multivariate analysis revealed that combined chemotherapy and radiation dose were prognostic factors for PFS (*p <* 0.05).

**Conclusions:**

Our center's data showed that the survival prognosis of locally advanced patients receiving RT and chemotherapy in China was consistent with international levels during the same period. Patients with a KPS score of 80 or higher, who had never smoked or received combined RT, had a more favorable prognosis than those with a KPS of less than 80, who had smoked, or only received RT. The combination of RT and chemotherapy, with a reasonable radiation dose, was the key to improving the therapeutic effect.

## Graphical abstract

The aim of this study was to investigate and report the data on treatment efficacy, toxicity, long‐term follow‐up results, and prognostic factors in LA‐NSCLC patients who underwent radical radiotherapy over the past decade in our center. Our center is one of the largest tumor treatment centers in China and has not previously reported such data.

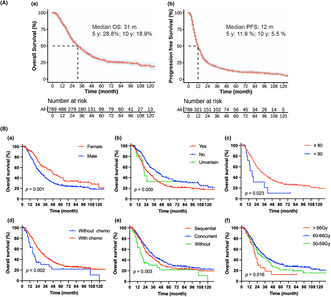

## 1 INTRODUCTION

Lung cancer is the leading cause of cancer‐related death worldwide.^1^ Globally, more than 2 million new cases were diagnosed, and 1.8 million deaths from lung cancer occurred in 2018 global cancer statistics, representing almost one in five deaths from cancer (18.4%).^1^ Non‐small cell lung cancer (NSCLC) accounts for 85% of lung cancer diagnoses, and locally advanced disease is present in one‐third of these cases at diagnosis.^2^, ^3^

Based on the RTOG 9410 study and other reports, the standard treatment for patients with inoperable stage III NSCLC was concurrent platinum‐based doublet chemoradiotherapy.^4^, ^5^ Previous prospective trials have demonstrated the superiority of platinum‐containing doublet chemotherapy combined with concurrent radiotherapy compared with sequential radiotherapy for patients with good performance status (PS)^4^, ^6^; sequential chemoradiotherapy (sCRT) is recommended for patients who cannot tolerate this regimen.^7^, ^8^ The 5‐year survival rate of concurrent chemoradiotherapy (cCRT) remains approximately 15%–30%.^2^, ^4^, ^9^ Although the optimal total dose and schedule of radical radiotherapy (RT) for stage III inoperable NSCLC remain controversial, the long‐term follow‐up results of the RTOG 0617 study have been reported. The median survival of the standard 60 Gy group was 28.7 months and 5‐year OS and PFS rates were 32.1% and 23%. But the high‐dose 74 Gy RT group was not beneficial in comparison to the standard dose.^9^ Currently, the standard RT dose range is 60–66 Gy.^9^, ^10^, ^11^ Recent findings from the PACIFIC clinical trial support the recommendation of cCRT followed by consolidation with durvalumab immunotherapy as the new standard of treatment modality as this improves survival.^12^ Median OS was 47.5 months with durvalumab versus 29.1 months with placebo. The estimated 5‐year OS rate was 42.9% with durvalumab versus 33.4% with placebo.^13^

There are numerous prognostic indicators for NSCLC treated with chemoradiotherapy; however, the stage at diagnosis is the critical determinant of prognosis.^14^, ^15^ Patients with the same clinical stage and course of treatment may have very different prognoses. Therefore, more studies are needed to determine the prognostic markers.

So far, there have been few reports on the survival results of radical RT for stage III lung cancer in a large sample of patients in China. From the RTOG 0617 experience, factors predictive of better OS on multivariable analysis include treatment at centers with higher enrollment volumes. Clinical trial accrual volume may represent the lung cancer clinical volume of the institution overall.^16^ Our center is one of the largest tumor treatment centers in China and has not previously reported such data. Although the current treatment of lung cancer has entered the immune/targeted therapy era, RT is still an indispensable radical treatment method. It is still necessary to review the survival data of the real‐world data on lung cancer RT, as a baseline reference and evidence‐based basis for subsequent research and comparison. The aim of this study is to investigate and report the data on treatment efficacy, toxicity, long‐term follow‐up results, and prognostic factors in locally advanced non‐small cell lung cancer (LA‐NSCLC) patients who underwent radical RT over the past decade in our center.

## 2 METHODS

### 2.1 Ethical approval

This study was conducted by the Declaration of Helsinki and the Good Clinical Practice guidelines. The institutional ethics review board approved the study protocol. Exemption from the participant's informed consent had been applied to the hospital ethics committee because of the retrospective nature of the study.

### 2.2 Patients and enrolment

**FIGURE 1:**
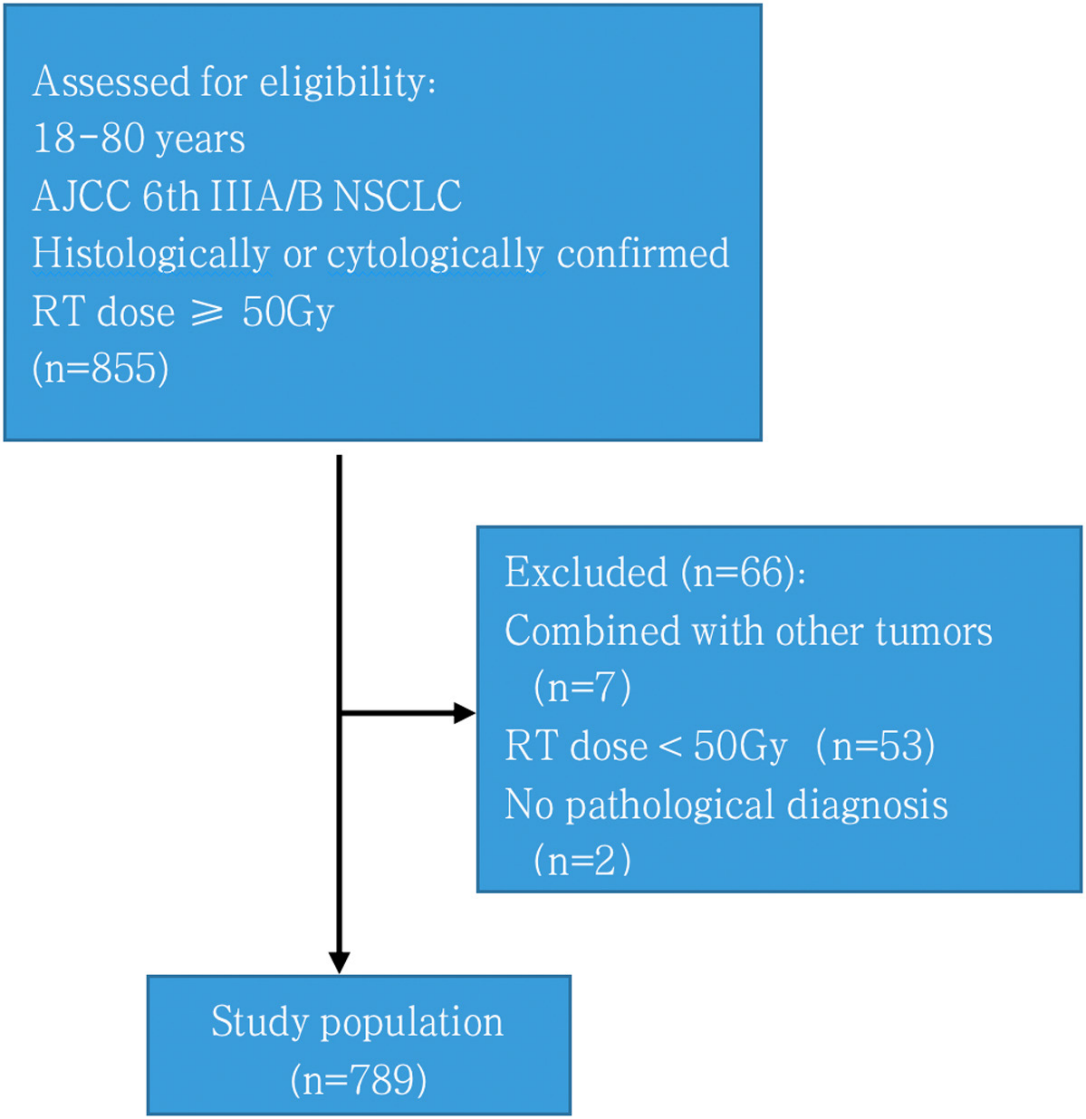
Flowchart of the study selection process. From a database of 855 patients, 789 with LA‐NSCLC treated in 2005–2015 were selected.

The study design was a single institutional, retrospective, observational study. In total, 855 patients with inoperable, LA‐NSCLC who underwent radical RT between January 2005 and December 2015 at our Cancer Center were enrolled in this study (Figure 1). The inclusion criteria were as follows: age, 18–80 years; histologically or cytologically confirmed diagnosis of NSCLC; stage IIIA or B according to the American Joint Committee on Lung Cancer's sixth version of the TNM classification the sixth edition of the American Joint Committee on Lung Cancer classification; and minimum RT dose of 50 Gy. The exclusion criteria were as follows: prior history of thoracic RT or surgery for malignant disease, coexistence with other malignant tumors, and radiation dose under 50 Gy. The enrolled patients had not used immunotherapy drugs. Finally, 789 patients were taken into account for the analysis.

### 2.3 Radiation therapy

All patients received a radical treatment dose of RT. Gross tumor volume was defined as primary tumor and lymph nodes that could be found. The clinical target volumes of the primary tumor and the involved lymph nodes were determined during RT planning by an additional margin (3–5 mm for lymph nodes and 5–8 mm for the primary tumor), and a further 5–10 mm margin was added to achieve the planning target volumes (PTV) according to local guidelines. Organs at risk included the lungs, heart, spinal cord, esophagus, and brachial plexus. A linear accelerator with 6MV x‐rays was used to deliver the treatment. The radiation regimen consisted of 2 Gy per fraction, five fractions per week, with a target dose of 50–77 Gy (median, 60.9 Gy) delivered either using three‐dimensional conformal radiotherapy (3D‐CRT) or intensity‐modulated radiotherapy (IMRT). The recommended prescribed dose was administered to 95% or more of the PTV. For 61.6% of patients, the total radiation dose was 60–66 Gy. IMRT was administered in 78.3% of the total patients, while 3D‐CRT was administered in 21.7%.

### 2.4 Chemotherapy

Of the 789 patients undergoing definitive RT, cCRT was delivered to 42%, sCRT to 49.3%, and RT alone to 8.7% (Table 1). In this study, the majority (92.7%, 307/331) of patients underwent cCRT containing platinum chemotherapy, and in 26.7% (82/307) of patients, a weekly treatment regimen was applied. The two most commonly used regimens are etoposide–cisplatin and carboplatin–paclitaxel.^8^, ^17^ The combination of platinum‐based chemotherapy and taxanes (paclitaxel or docetaxel) was used most frequently with cCRT (42.9%). Various regimens were used in patients undergoing sCRT (49.3%) reflecting the flexibility in clinical practice permitted by institutional clinical protocols. The most commonly used treatment combinations included: docetaxel plus cisplatin, vinorelbine plus cisplatin, pemetrexed plus cisplatin, and gemcitabine plus cisplatin.

**TABLE 1:** Patient baseline characteristics (*n* = 789) and univariate analysis of clinical factors influencing the occurrence of radiation pneumonitis.

Clinical characters	Number	Percentage (%)	Acute radiation pneumonitis (%)	*χ* ^2^	*p*‐value	Chronic radiation pneumonitis (%)	*χ* ^2^	*p*‐value
Sex				0.507	0.504		0.132	0.774
Male	638	80.9	218 (34.2)			214 (33.5)		
Female	151	19.1	47 (31.1)			53 (35.1)		
Age (years)				0.379	0.546		1.162	0.292
≥60	423	53.6	138 (32.6)			136 (32.2)		
<60	366	46.4	127 (34.7)			131 (35.8)		
Smoking status				30.785	<0.001		23.658	<0.001
Current/former	455	57.7	176 (38.7)			162 (35.6)		
Never	285	36.1	89 (31.2)			104 (36.5)		
Unknown	49	6.2	0 (0)			1 (2.0)		
KPS score				0.831	0.483		0.783	0.483
≥80	768	97.3	258 (33.3)			258 (33.6)		
<80	21	2.7	9 (42.9)			9 (42.9)		
Pathology type				4.299	0.118		1.071	0.582
Adenocarcinoma	292	37	90 (30.8)			98 (33.6)		
Squamous cell carcinoma	323	40.9	122 (37.8)			115 (35.6)		
NOS	174	22.1	53 (30.5)			54 (31.0)		
Stage				0.110	0.760		0.210	0.648
IIIA	328	41.6	108 (32.9)			114 (34.8)		
IIIB	461	58.4	157 (34.1)			153 (33.2)		
EGFR and ALK status				2.071	0.360		13.401	0.001
EGFR(+) or ALK(+)	62	7.9	19 (30.6)			31 (50.0)		
EGFR(−) and ALK(−)	87	11	35 (40.2)			38 (43.7)		
Unknown	640	81.1	211 (33.0)			198 (30.9)		
Weight loss				9.164	0.003		0.294	0.628
<5%	704	89.2	224 (31.8)			236 (33.5)		
≥5%	85	10.8	41 (48.2)			31 (36.5)		
Combined chemotherapy				0.237	0.689		2.860	0.110
Yes	720	91.3	240 (33.3)			250 (34.7)		
No	69	8.7	25 (36.2)			17 (24.6)		
Chemotherapy modality				1.042	0.601		8.532	0.014
Concurrent	331	42	116 (35.0)			130 (39.3)		
Sequential	389	49.3	124 (31.9)			120 (30.8)		
Without	69	8.7	25 (36.2)			17 (24.6)		
Radiation dose				4.368	0.113		2.011	0.364
≥66 Gy	40	5.1	8 (20)			10 (25.0)		
60–66 Gy	616	78.1	216 (35.1)			215 (34.9)		
<60 Gy	133	16.9	41 (30.8)			42 (31.6)		
Radiation modality				13.975	<0.001		14.520	<0.001
3D‐CRT	171	21.7	37 (21.6)			37 (21.6)		
IMRT	618	78.3	228 (36.9)			230 (37.2)		
Obstructive pneumonitis				26.243	<0.001		24.897	<0.001
No	686	86.9	241 (35.1)			246 (35.9)		
Yes	52	6.6	23 (44.2)			20 (38.5)		
Unknown	51	6.5						
Interstitial pneumonitis				25.402	<0.001		26.547	<0.001
No	716	90.7	254 (35.5)			261 (36.5)		
Yes	22	2.8	10 (45.5)			5 (22.7)		
Unknown	51	6.5						

Abbreviation: NOS, not otherwise specified.

### 2.5 Follow‐up and evaluation

The patients were followed up via reviews of their medical records, imaging data, and routine phone calls. According to the case records, patients underwent regular follow‐ups with their treating physician during treatment. After the completion of treatment delivery, patients were followed up for 1–3 months. Follow‐up was at 3‐monthly intervals for the first year, with assessment checks and imaging as necessary; 6‐monthly intervals for the second and third years; and every 6–12 months after the first 3 years. The last follow‐up day was December 31, 2021. The primary outcomes were overall survival (OS) and progression‐free survival (PFS). The secondary outcomes were treatment efficacy and treatment‐related toxicities. Efficacy was evaluated according to the Response Assessment in RECIST 1.0. The overall clinical benefit rate was the sum of complete response, partial response, and stable disease. The ultimate toxicity grade was defined as the highest among acute toxicities during radiation and late toxicities during follow‐up visits. Radiation‐related toxicity was identified and assessed according to the Common Terminology Criteria for Adverse Events (CTCAEv3.0).^18^ In clinical practice, acute or early radiation reactions occur within 3 months after the start of radiation therapy. The radiation reaction that occurs 3 months later is called chronic or late radiation reaction.^19^

### 2.6 Statistical analysis

OS and PFS analyses were performed. The OS was calculated from the start date of RT or chemotherapy to the date of death due to any cause. OS was censored on the last date known to be alive for these patients who were not known to have died as of the lock date. PFS was calculated from the initiation date of treatment to the first date of progressive disease, or death for any reason. For these patients who were not known to have died or had an objective progressive disease as of the data lock date, PFS was censored on the date of the last objective progression‐free disease assessment. OS and PFS survival curves were calculated using the Kaplan–Meier method and compared using the log‐rank test. The variables that showed statistical significance (*p <* 0.05) in the univariate analysis were included in the multivariate analysis. Stepwise multivariate analysis was performed using the Cox regression model and forward logistic regression. The chi‐square test was used to compare the clinical characteristics between groups of patients with and without acute or chronic radiation pneumonitis. SPSS statistics software (IBM version 26) and GraphPad Prism 10 were used to process the data and generate the graphs. Statistical significance was set at *p <* 0.05.

## 3 RESULTS

### 3.1 Clinical characteristics

The baseline patient demographics, clinical, and treatment characteristics are summarized (Table 1). The median age at diagnosis was 61 years (range: 20–87 years), and 19.1% of patients were female. Stages IIIA and IIIB accounted for 41.6% and 58.4% of cases, respectively. Squamous cell carcinoma accounted for 40.9% of all cases, adenocarcinoma for 37%, and others (not otherwise specified) for 22.1%.

### 3.2 Survival and treatment failure

**FIGURE 2:**
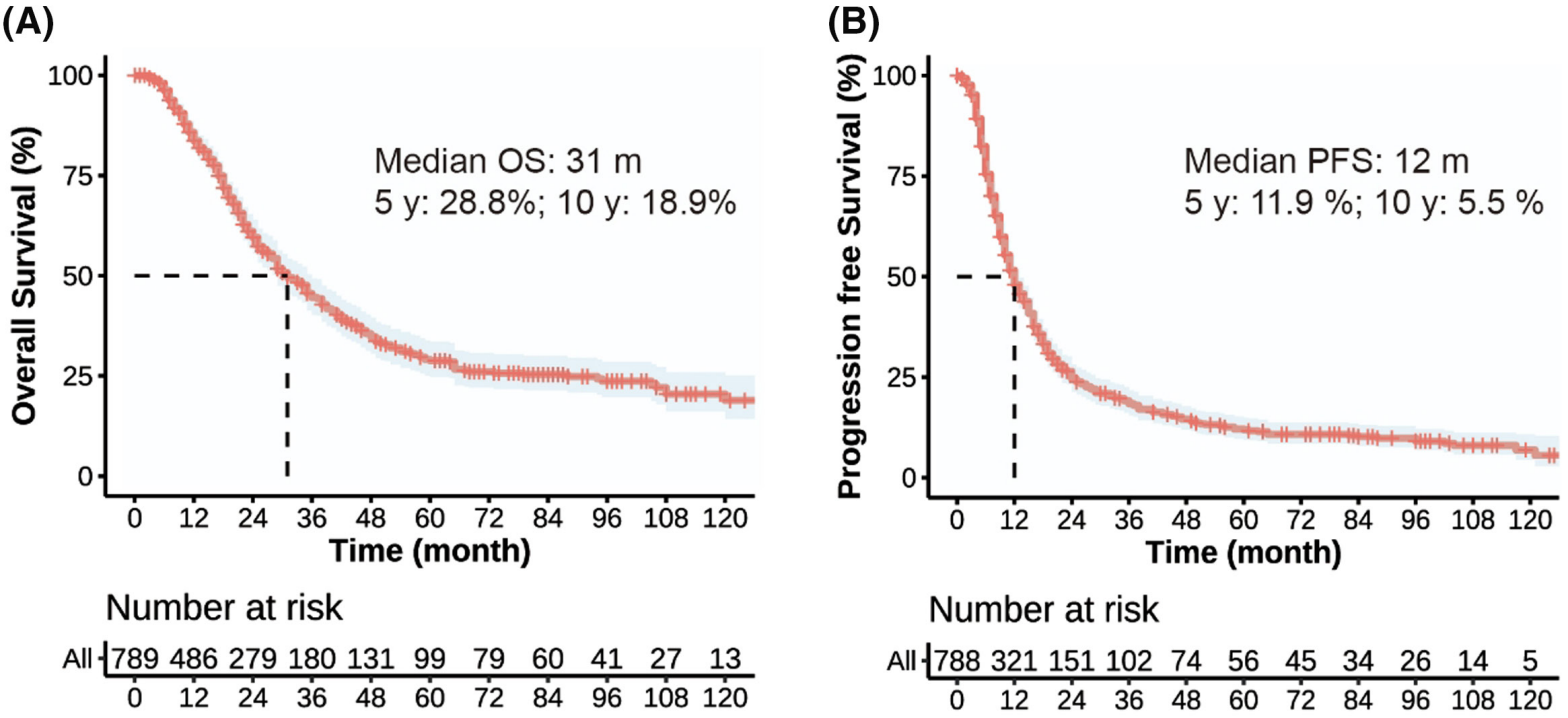
Kaplan–Meier survival analyses for locally advanced non‐small cell lung cancer treated with definitive radiotherapy. (A) Median overall survival of 31 months, (B) median progression‐free survival of 12 months.

**FIGURE 3:**
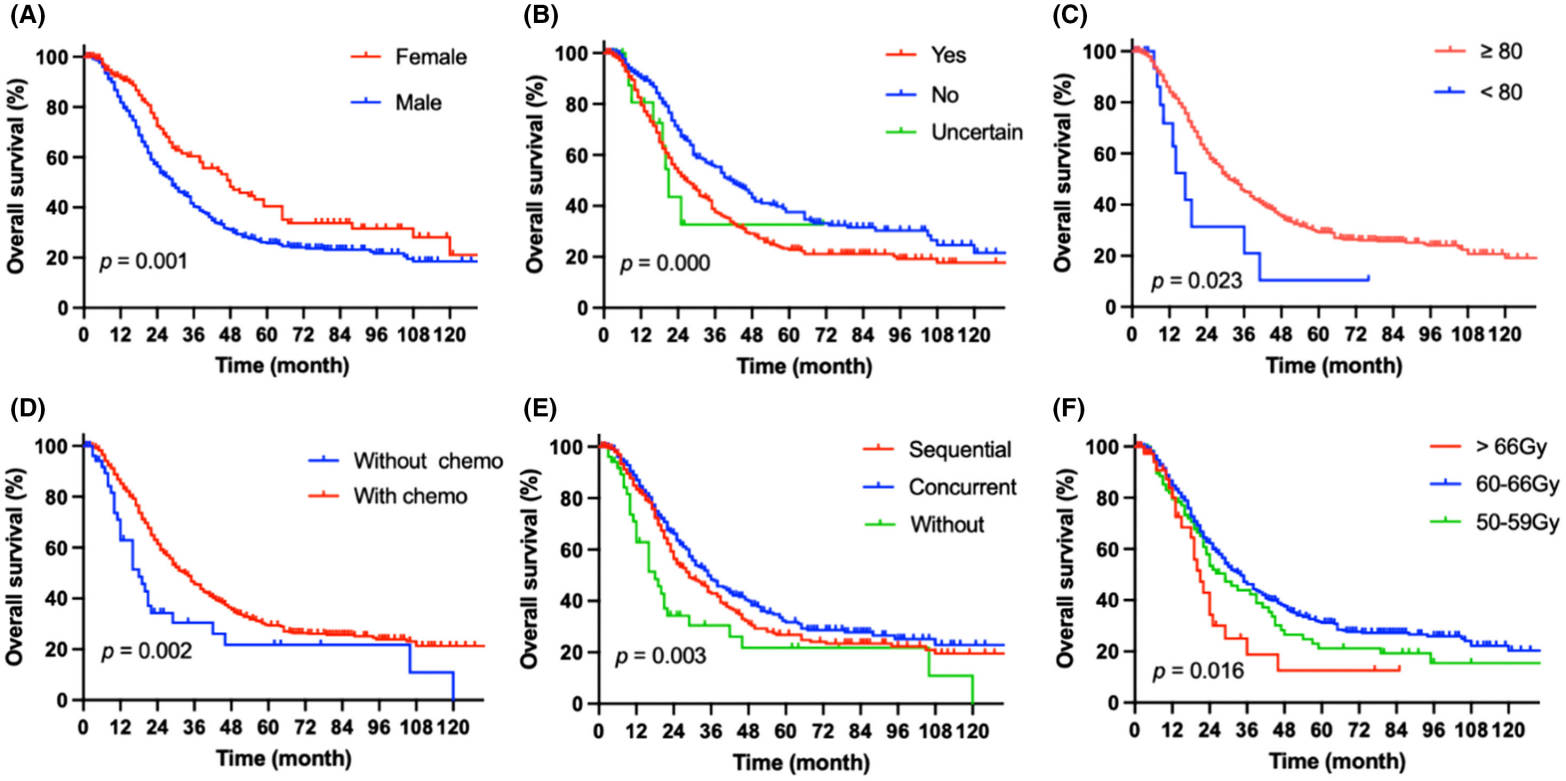
Graphs A–F depicting clinical parameters that affect overall survival in univariate analysis (A, sex; B, smoking status; C, KPS score; D, combined chemotherapy; E, chemotherapy modality; F, radiation dose).

**FIGURE 4:**
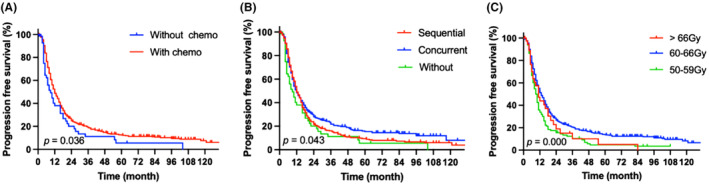
Graphs A–C depicting clinical parameters that affect progression‐free survival in univariate analysis (A, combined chemotherapy; B, chemotherapy modality; C, radiation dose).

**FIGURE 5:**
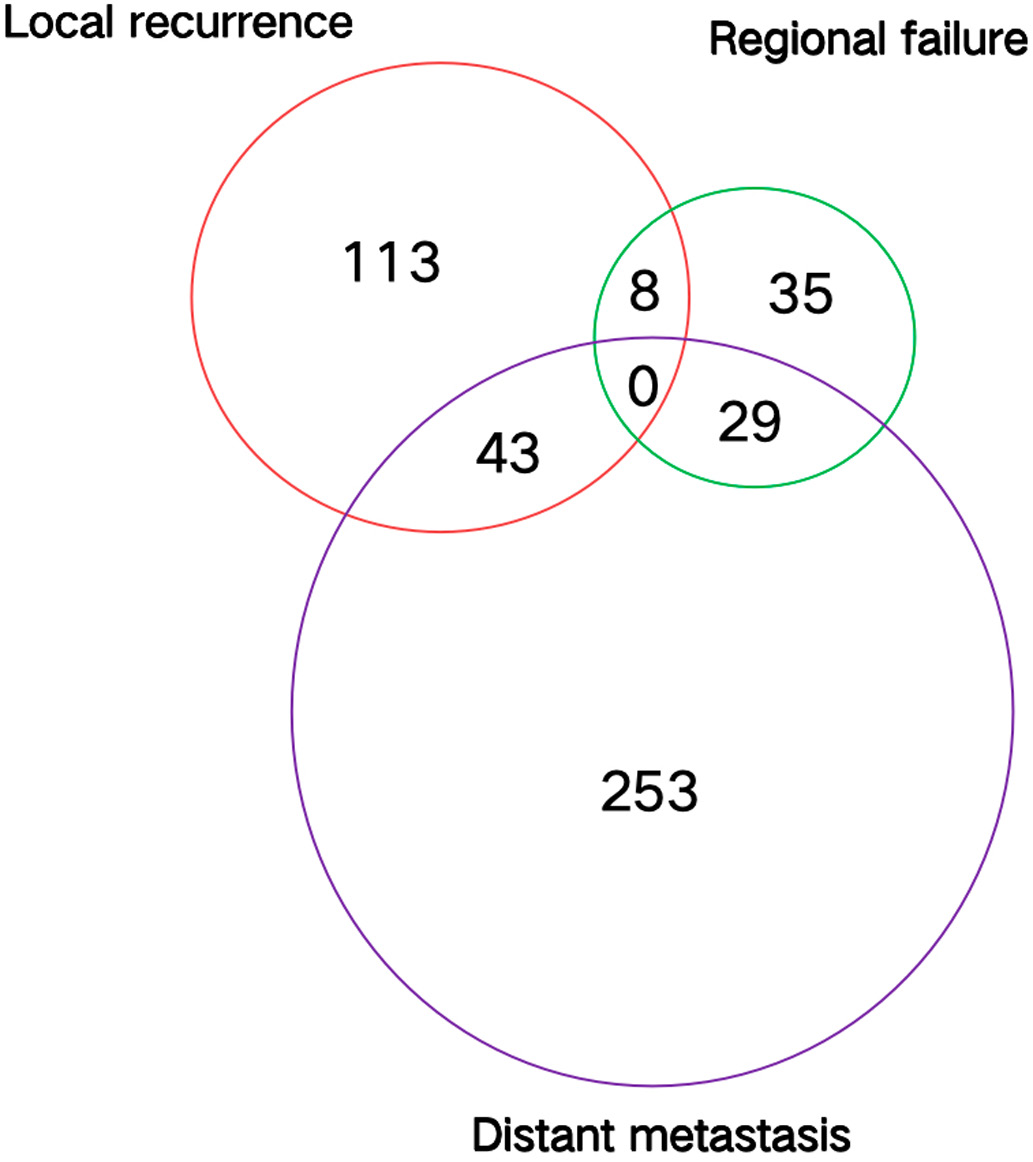
Patterns of disease failure as the first diagnosis for 789 locally advanced non‐small cell lung cancer treated with radiotherapy.

To assess short‐term efficacy, the patients were reexamined 1–3 months after RT. Among the 789 patients, there were 21 cases of complete response (2.7%), 513 cases of partial response (65%), 101 cases of stable disease (12.8%), and 154 cases of progressive disease (19.5%). By the end of the follow‐up period, there were 365 overall deaths and 576 cases of recurrence, metastasis, or death. The median follow‐up time was 42 months. The median survival time of the patients was 31 months and the median PFS time was 12 months (Figure 2). The 1‐, 2‐, 5‐, and 10‐year OS rates were 83.7%, 59.5%, 28.8%, and 18.9%, respectively. The median survival time of patients with stage IIIA NSCLC was 31 months, whereas those with stage IIIB NSCLC had a median survival time of 30 months (*p =* 0.998). In addition, the 1‐, 2‐, 5‐, and 10‐year PFS rates were 48%, 24.5%, 11.9%, and 5.5%, respectively (Figures 2, 3, 4). There were 365 deaths, including 7 non‐tumor‐related deaths and 16 radiation pneumonia‐related deaths. At the last follow‐up, 499 patients had recurrences or distant metastases. Of these, 164 had local recurrence, 72 had regional failure, and 325 had distant metastasis when first diagnosed with disease progression (Figure 5). The first 2 years after RT were the peak period of recurrence, and a small proportion (7%) of patients still survive for a long time after recurrence. Local failure was defined as disease progression within the original tumor. Regional failure was defined as disease progression in regional lymph nodes.

### 3.3 Treatment‐related toxicities

Rates of ≥grade 3 acute radiation pneumonitis or esophagitis were 7.6% and 1.9%, respectively. Rates of ≥grade 3 chronic radiation pneumonitis and esophagitis were 11% and 0.4%, respectively (Table 2). The incidence rate of grade 5 chronic radiation pneumonia among patients was 2.0% (16/789). Treatment‐related hematological toxicities during RT were shown (Table 2). The occurrence of radiation pneumonia was related to clinical factors and physical dose parameters, and this study only explored clinical‐related factors (Table 1). In univariate analysis, clinical factors related to the occurrence of acute radiation pneumonia included smoking status, weight loss, radiation modality, obstructive pneumonitis, and interstitial pneumonitis; The clinical factors associated with the occurrence of chronic radiation pneumonia included smoking status, epidermal growth factor receptor (EGFR) and anaplastic lymphoma kinase (ALK) status, chemotherapy modality, radiation modality, obstructive pneumonitis, and interstitial pneumonitis.

**TABLE 2:** Treatment‐related pulmonary, esophageal, and hematological toxicity.

Adverse reactions	Adverse reaction classification				
	Grade 1 (%)	Grade 2 (%)	Grade 3 (%)	Grade 4 (%)	Grade 5 (%)
Acute toxicity					
Radiation pneumonitis	100 (12.7)	105 (13.3)	60 (7.6)		
Radiation esophagitis	207 (26.2)	52 (6.6)	15 (1.9)		
Leukopenia	193 (24.5)	182 (23.1)	89 (11.3)	20 (2.5)	
Neutropenia	105 (13.3)	99 (12.5)	65 (8.2)	37 (4.7)	
Thrombocytopenia	118 (15.0)	67 (8.5)	19 (2.4)	14 (1.8)	
Hemoglobinemia	188 (23.8)	58 (7.4)	19 (2.4)	4 (0.5)	
Chronic toxicity					
Radiation pneumonitis	75 (9.5)	105 (13.3)	70 (8.9)	1 (0.1)	16 (2)
Radiation esophagitis	6 (0.8)	34 (4.3)	2 (0.3)	1 (0.1)	0 (0)

### 3.4 Prognostic analysis

The following factors were analyzed for their impact on survival: sex, age, smoking status, PS, pathology type, stage, EGFR or ALK status, weight loss, combined chemotherapy, chemotherapy modality, and radiation dose. Univariate analysis identified sex, smoking status, Karnofsky Performance Status (KPS) score, combined chemotherapy, chemotherapy modality, and radiation dose as significant predictive factors for OS (Figures 3, Table 3). The multivariate analysis took these variables into account. Smoking status (*p =* 0.002), KPS score (*p =* 0.036), and combined chemotherapy (*p =* 0.003) were independent predictors of OS in the multivariate analysis. However, the significant predictors of PFS were combination chemotherapy (*p =* 0.014) and radiation dose (*p =* 0.004) (Table 3). According to our findings, pathological type and tumor stage (stage IIIA vs. IIIB) did not affect survival.

**TABLE 3:** Univariate and multivariate analysis of overall survival and progression‐free survival of locally advanced non‐small cell lung cancer.

Variables	Univariate		Multivariate		Univariate		Multivariate	
	mOS	*p*‐value	HR (95% CI)	*p*‐value	mPFS	*p*‐value	HR (95% CI)	*p*‐value
Sex		0.001				0.236		
Male	29				12			
Female	48				14			
Age (years)		0.066				0.091		
≥60	37				11			
<60	29				14			
Smoking status		0.000	0.730	0.002		0.241		
Current/former	27		0.597–0.893		11			
Never	42				14			
Unknown	21				19			
KPS score		0.023	1.966	0.036		0.086		
≥80	31		1.046–3.695		12			
<80	17				6			
Pathology type		0.071				0.107		
Adenocarcinoma	38				11			
Squamous cell carcinoma	29				15			
NOS	27				10			
Stage		0.998				0.142		
IIIA	31				14			
IIIB	30				11			
EGFR and ALK status		0.079				0.586		
EGFR(+) or ALK(+)	52				12			
EGFR(−) and ALK(−)	25				11			
Unknown	30				13			
Weight loss		0.093				0.197		
<5%	33				12			
≥5%	25				9			
Combined chemotherapy		0.002	1.750	0.003		0.036	1.471	0.014
Yes	33		1.203–2.545		12		1.081–2.002	
No	18				9			
Chemotherapy modality		0.003				0.043		
Concurrent	35				12			
Sequential	29				12			
Without chemo	18				9			
Radiation dose		0.016				0.000	0.754	0.004
≥66 Gy	21				11		0.622–0.915	
60–66 Gy	34				13			
<60 Gy	29				9			
Radiation modality		0.361				0.503		
3D‐CRT	29				12			
IMRT	32				12			

## 4 DISCUSSION

There is considerable research on patients with LA‐NSCLC treated with definitive RT.^9^, ^14^, ^20^ Big data analysis is increasingly valued for its contribution to precision medicine, clinical decision‐making, and quality enhancement. Some research conducted by large institutions summarized the current status of the effectiveness and safety of LA‐NSCLC radical RT in patients in clinical practice before the approval of Durvalumab. In the RTOG 0617 study, the conventional 60 Gy group's median survival time was 28.7 months.^9^ The KINDLE study, a retrospective, international, multicenter, non‐interventional study, revealed the diversity of treatment options and clinical outcomes for patients with stage III NSCLC in the pre‐immunotherapy era. The median PFS and OS in the overall population were 12.5 and 34.9 months, respectively.^14^ The UNIVERSE‐ROOT study captured real‐world patterns of the ten‐year journey of patients with stage III NSCLC. The median OS was 30.3 months for patients treated with radical intention.^20^ To our knowledge, there was limited research data on large‐scale stage III lung cancer radical RT reported in China. Chinese Academy of Medical Sciences and Peking Union Medical College reported that median survival times were 23.3 months in the etoposide plus cisplatin arm versus 20.7 months in the paclitaxel plus carboplatin arm in the concurrent thoracic RT trial.^21^ Our retrospective investigation included a substantial number of patients; the median OS was 31 months, which compares favorably with previously reported data. The prognosis of patients with lung cancer is influenced by various factors, which can be categorized as tumor‐related, patient‐related, and treatment‐related variables.

Globally, cases diagnosed as early and LA‐NSCLC include a greater proportion of men than women (77.2% vs. 22.8%).^15^ According to some reports, sex does not influence survival, although trials have shown that long‐term survival rates are better in women.^14^, ^22^, ^23^ Our study supports this hypothesis. Our study included more men (80.9%) than women (19.1%). The median survival time was 48 months for women and 29 months for men and the difference between the two groups was significant (*p =* 0.001). Russell et al. retrospectively analyzed 184 patients undergoing radical RT at the Ottawa Hospital Cancer Center for stage IIIB NSCLC and found that greater OS rates were identified in women.^22^ Multivariate analysis of 4954 patients with stage IIIB and IV NSCLC from the National Hospital Study Group for Lung Cancer in Osaka, Japan, found that OS was significantly longer in women than in men (*p <* 0.0001).^23^

Smoking is also known to be one of the critical, independent predictors of reduced survival.^14^, ^15^ Lodovici et al. found that tobacco‐containing carcinogen nitrosamines can result in mutations in the c‐Met gene and changes in the tumor suppressor genes Rb, P53, and other gene loci, which may eventually induce the development of lung cancer.^24^ Long‐term smokers tend to be male, and most have emphysema and chronic bronchitis, which impair lung function and increase the risk of developing pulmonary infections and malignant pleural effusions.^25^ Using a large database, Kawaguchi et al. found that never‐smoking status was a marginal but significant prognostic factor in patients with advanced NSCLC compared to ever‐smokers.^23^ Adenocarcinoma histology was clearly linked to increased survival in never‐smokers, but not in ever‐smokers. This may be explained by EGFR mutations, which were observed more frequently in adenocarcinoma in never‐smokers than in ever‐smokers.

The relationship between PS and prognosis has been demonstrated by many investigators, with PS being increasingly acknowledged as a significant adverse prognostic factor.^11^, ^14^, ^26^ In a phase III trial, good PS was a positive prognostic factor for survival. The median OS for the Eastern Cooperative Oncology Group (ECOG) 0–1 arm was 30.1 months, compared to 16.4 months for the ECOG 2 arm (*p* < 0.001).^27^ We also confirmed that PS (KPS score) prior to therapy had a significant impact on survival.

A combination of RT and platinum‐based chemotherapy is generally recommended for LA‐NSCLC. Chemotherapy is supposed to lower the possibility of distant metastasis, while RT is supposed to maintain localized control. Additionally, chemotherapeutic agents may enhance radiosensitivity and boost the efficiency of radiation therapy. To date, several randomized clinical studies and meta‐analyses have revealed improved survival outcomes with cCRT compared with sCRT or RT alone in patients with unresectable stage III NSCLC in good general condition with minimal weight loss.^4^, ^5^, ^28^ For weak patients who cannot tolerate cCRT, sCRT or RT alone are suitable because of the higher toxicity associated with cCRT.^29^, ^30^ In controlled and real‐world studies, the median OS of patients with stage III NSCLC undergoing cCRT varies from 15 to 29 months.^4^, ^31^ A retrospective multicenter review found that elderly patients (70 years old and older) with stage III NSCLC treated with cCRT did not show improved OS compared to those treated with sCRT or RT alone.^32^ The largest reported cohort of elderly patients from the National Cancer Database revealed a 9% reduction in the risk of death for those treated with sCRT compared to cCRT.^29^ In our study, the median survival times for cCRT, sCRT and RT alone were 35, 29, and 18 months, respectively (*p =* 0.003) (Table 3), when we conducted subgroup analysis by age of 60 years old. In patients 60 years old and or older, there was no statistically significant difference in the impact of treatment modality (cCRT, sCRT, or RT alone) on OS (*p =* 0.073); but among those under 60 years old, cCRT was superior to sCRT and RT alone (*p* < 0.01). Therefore, cCRT may not be the most effective treatment modality for elderly patients.

Currently, the European Society for Medical Oncology and other studies recommend conventionally fractionated CRT at 60–66 Gy.^11^, ^26^ A large retrospective analysis of the National Cancer Database assessed the role of dose escalation in the setting of stage III NSCLC. Dose escalation above 60 Gy was linked to a better OS in this cohort of patients treated with chemoradiotherapy. A benefit plateau was observed, with no additional improvement in OS with a higher dose (71 Gy) compared with 66–70 Gy. These findings support further research on the role of intermediate‐dose radiation.^33^ The long‐term follow‐up results of the RTOG 0617 study revealed that the median survival of the standard 60 Gy group was 28.7 months. In contrast, the median survival of the high‐dose 74 Gy group was 20.3 months. The RTOG 0617 trial failed to prove improved outcomes with the addition of high‐dose RT and cetuximab.^9^ The present study yielded similar results: patients receiving radiation doses of 60–66 Gy had better OS and PFS survival than those in the groups with radiation doses above 66 Gy or below 60 Gy. Multivariate Cox analysis revealed that radiation dose was associated with PFS but not OS prognosis (Table 3).

The effect of IMRT and 3D‐CRT techniques on prognosis remains disputed. Historically, LA‐NSCLC has been treated using 3D‐CRT. IMRT provides the theoretical ability to increase the therapeutic ratio compared to 3D‐CRT. There is no randomized research comparing IMRT to 3D‐CRT; all comparative studies were retrospective,^34^ except for the secondary analyses of the RTOG 0617. Even in the RTOG 0617 trial, the choice of RT technique was not randomized, introducing the same biases present in retrospective studies.^35^ The study comparing the outcomes of IMRT and 3D‐CRT found no differences in two‐year OS, PFS, local failure, and distant metastasis‐free survival between them. IMRT was linked to lower rates of more than grade 3 pneumonitis (7.9% vs. 3.5%, *p =* 0.039) and a reduced risk in adjusted analyses (odds ratio, 0.41; 95% CI, 0.171–0.986; *p =* 0.046). IMRT also resulted in lower heart dose (*p <* 0.05). However, the IMRT group had larger and more advanced tumors; therefore, it is yet unclear which radiation treatment technique gives advantages for long‐term effects.^36^ A meta‐analysis of 10 retrospective studies revealed that 3D‐CRT and IMRT exhibited comparable OS rates (HR = 0.96, *p =* 0.477).^34^ However, IMRT significantly reduces the risk of radiation pneumonitis and increases the risk of radiation esophagitis compared with 3D‐CRT. According to our study, the adoption of 3D‐CRT and IMRT techniques did not significantly affect OS or PFS. When compared to 3D‐CRT, IMRT has more grade II and higher acute (*p =* 0.0017) and chronic (*p <* 0.0001) radiation pneumonia cases.

Although radiation esophagitis is a common toxicity associated with RT for lung cancer, life‐threatening radiation esophagitis is rare. In this study, no lethal esophagitis occurred. Symptomatic radiation esophagitis improves with nutritional support.^37^ Radiation pneumonitis is the most critical dose‐limiting toxicity of RT for lung cancer. The treatment‐related acute pulmonary toxicity rate in patients undergoing chemoradiotherapy varies from 4.8% to 47% across different studies. The risk of radiation pneumonitis is also related to different chemotherapy drugs or chemotherapy regimens used in combination with RT.^38^ In this study, the rate of grade 3 acute radiation pneumonitis was 7.6%, and the rate of ≥grade 3 chronic radiation pneumonitis was 11%. The incidence rate of grade 5 chronic radiation pneumonia among patients was 2.0% (16/789), and 16 cases were found to be male, including 12 cases of squamous cell carcinoma. The incidence rate of KPS ≥80 group was 1.8%, and the incidence rate of KPS < 80 was 9.5%. However, the two groups had no significant statistical difference (*p =* 0.065). Compared with other clinical factors of the population without grade 5 pneumonia, no significant influencing factors were found in the chi‐square test. Compared with similar studies, the incidence of grade 5 pneumonia in the RTOG 0617 study was lower than 1% in both the 60 Gy and the 60 Gy plus cetuximab groups.^10^ Stage IIIA accounted for over 60%, while IIIB accounted for less than 40%. A multicenter phase III trial conducted by the Cancer Hospital of the Chinese Academy of Medical Sciences aimed to compare the efficacy of concurrent radiation therapy with etoposide/cisplatin or carboplatin/paclitaxel in patients with stage III NSCLC.^21^ Among the 9 cases (4.7%) of acute treatment‐related deaths, all were caused by radiation pneumonitis, and there was no clear evidence of differences between the arms in the rates of these grade 5 toxicities. In this study, IIIA accounted for 25%, with a higher proportion of IIIB patients, accounting for 75%.

Our data were prior to the phase III PACIFIC trial results, and immunotherapy was not the mainstream treatment option then. The limitations of the present study include biases associated with the retrospective study design in a real‐world setting. Initially, data collection was limited by the accuracy of existing medical records, resulting in missing data, as some patients may have been lost to routine clinical follow‐up. Also, RT toxicity may have been underestimated, resulting in an inaccurate comparison of radiation response between 3D‐CRT and IMRT. The time span was long and there was heterogeneity in the treatment strategies. Further, the number of patients undergoing genetic testing was low; therefore, the number of cases that can be analyzed is limited. The TNM staging system for lung cancer in clinical use has been updated to the eighth edition. As our patients were enrolled earlier, we applied the sixth edition of the staging system for consistency across the study population. Nonetheless, the strength of this study is the long study period and the large sample size, which makes the sample representative of the study area and the temporal changes occurring over the last decade. A prospective observational cohort study is required to ensure accurate and detailed data collection and validate these findings.

## 5 CONCLUSION

In conclusion, our study revealed the variety of treatment practices and outcomes in stage III NSCLC in a real‐world setting before the approval of immunotherapy as an option. The practices observed in our study reflected the state of lung cancer treatment over the last 10‐year study period. Smoking status, KPS score, and combined chemotherapy were confirmed to be the most important prognostic factors in stage III lung cancer treated with definitive RT. Combined chemotherapy and radiation dose were independent prognostic factors for PFS. The combination of RT and chemotherapy, with a reasonable radiation dose, was the key to improving the therapeutic effect.

## AUTHOR CONTRIBUTIONS

**Hong Zhu:** Conceptualization (equal); data curation (equal); investigation (equal); methodology (equal); project administration (equal); resources (equal); software (lead); writing – original draft (lead). **Yi Xu:** Data curation (equal); investigation (equal). **Huiquan Gao:** Data curation (equal); investigation (equal). **Xingwen Fan:** Conceptualization (equal); formal analysis (equal); software (equal); supervision (equal); validation (equal). **Ming Fan:** Resources (equal). **Kuaile Zhao:** Resources (equal). **Huanjun Yang:** Resources (equal). **Zhengfei Zhu:** Resources (equal). **Kailiang Wu:** Conceptualization (equal); funding acquisition (lead); methodology (equal); project administration (equal); resources (equal); supervision (lead); writing – review and editing (lead).

## CONFLICT OF INTEREST STATEMENT

The authors declare no conflicts of interest.

## ETHICS STATEMENT

The protocol of this study was approved by the Fudan University Cancer Hospital Institutional Review Board (SCCIRB) and conducted in accordance with the Principles of the Helsinki Declaration. Written inform consent was waived by the Fudan University Cancer Hospital Institutional Review Board because of the retrospective nature of the study.

## Data Availability

The data that support the findings of this study are available on request from the corresponding author. The data are not publicly available due to privacy or ethical restrictions.
